# Milan Criteria and UCSF Criteria: A Preliminary Comparative Study of Liver Transplantation Outcomes in the United States

**DOI:** 10.1155/2012/253517

**Published:** 2012-08-22

**Authors:** Supriya S. Patel, Amanda K. Arrington, Shaun McKenzie, Brian Mailey, Michelle Ding, Wendy Lee, Avo Artinyan, Nicholas Nissen, Steven D. Colquhoun, Joseph Kim

**Affiliations:** ^1^Department of Surgery, University of Southern California, Los Angeles, CA 90098, USA; ^2^Department of Surgery, City of Hope Comprehensive Cancer Center, Duarte, California, CA 91010, USA; ^3^Department of Surgery, University of Kentucky, Lexington, KY 40506-9983, USA; ^4^Department of Surgery, Baylor College of Medicine, Houston, TX 77030, USA; ^5^Division of Transplantation, Department of Surgery, Cedars-Sinai Medical Center, Los Angeles, CA 90048, USA

## Abstract

The application of orthotopic liver transplantation (OLT) for patients with hepatocellular cancer (HCC) necessitates highly selective criteria to maximize survival and to optimize allocation of a scarce resource. The objective of this study was to compare the outcomes of OLT for HCC in patients transplanted under Milan and UCSF criteria. The United Network of Organ Sharing (UNOS) database was queried for patients who had undergone OLT for HCC from 2002 to 2007, and 1,972 patients (Milan criteria, *n* = 1, 913; UCSF criteria, *n* = 59) were identified. Patients were stratified by pretransplant criteria (Milan versus UCSF), and clinical and pathologic factors and overall survival were compared. There were no differences in age, gender, diabetes mellitus, body mass index, and hepatitis B, or C status between the two groups. Overall survival was similar between the Milan and UCSF cohorts (1-, 2-, 3-, and 4-year survival rates: 88%, 81%, 76%, and 72% versus 91%, 80%, 68% and 51%, respectively, *P* = 0.21). Although the number of patients within UCSF criteria was small, our results nevertheless suggest that patients with HCC may have equivalent survival when transplanted under Milan and UCSF criteria. Long-term followup may better determine whether UCSF criteria should be widely adopted.

## 1. Introduction

The incidence of hepatocellular carcinoma (HCC) has tripled in the United States during the past three decades with an annual increase of 4.5% [1]. Additionally, the mortality rates associated with this disease have continued to rise [1, 2]. The poor survival rates for HCC are in part due to the coexistence of advanced liver disease, which limits treatment options. Currently, orthotopic liver transplantation (OLT) has proven to be an excellent therapeutic option for long-term survival in patients presenting with early-stage HCC. In order to facilitate equitable allocation of donor livers, the United Network for Organ Sharing (UNOS) was established. Under UNOS guidelines, livers are appropriated based on a waiting list, in which patients are rank-ordered by their pretransplant mortality risk [3]. Unfortunately, relatively few eligible patients with HCC patients undergo OLT due to organ shortage. Each year, there are many more patients on the UNOS waitlist than there are organs available for transplantation [4]. Therefore, optimal allocation of organs is necessary for liver transplantation.

 When OLT was first proposed as a treatment for HCC, only patients who were unresectable due to tumor burden or other underlying liver dysfunction were selected as candidates [5]. As a result, patients who underwent OLT exhibited low survival and high recurrence rates after transplantation [6, 7]. These discouraging outcomes eventually resulted in refined selection criteria, such as those first proposed by Mazzaferro et al. in 1996 [8]. These criteria, known as the Milan criteria, have significantly improved survival. However, a subsequent report by Yao et al. indicated that the Milan criteria may be too restrictive [9]. Patients transplanted under more liberal selection criteria, designated as the UCSF criteria, and had outcomes comparable to those within Milan criteria. These data suggested that expansion of conventional criteria could broaden the patient pool for OLT without affecting the oncologic outcomes of OLT for HCC [9].

Despite an increase in organ donation over the last two decades, the number of patients awaiting liver transplantation has greatly exceeded the organ supply. More than 15,000 patients were listed for OLT in 2008, yet only approximately 5000 patients underwent OLT that year [4]. Appropriate selection of patients with HCC is therefore necessary to optimize the allocation of these organs. In light of this, the expansion of OLT criteria for HCC needs to be carefully weighed with regards to the limited organ supply and the outcomes of patients undergoing OLT with tumors outside of Milan criteria should be closely examined. The UNOS database has yet to be queried for evaluation of liver transplantation outcomes based on selection criteria. In this paper, we have used the UNOS database to compare the short-term survival outcomes of patients who underwent OLT for HCC within Milan criteria versus those undergoing OLT for HCC outside of Milan criteria, but within UCSF criteria. We hypothesized that patients transplanted within Milan criteria and those transplanted outside of Milan criteria but within UCSF criteria had equivalent outcomes.

## 2. Patients and Methods

### 2.1. UNOS Registry

Registered under the United States Department of Health and Human Services, UNOS maintains a database with detailed information about transplants performed in United States transplant centers. Via an internet-based database system known as UNet, this database application provides demographic, clinical, and pathologic data for all transplanted candidates and recipients. After obtaining approval for our study from UNOS and the City of Hope's institutional review board (IRB), the UNOS database was queried for patients who underwent OLT for HCC from 2002 through 2007. The year 2002 was selected as the first year of our study, since it was the first year following publication of the UCSF criteria [9]. Size criteria for Milan or UCSF criteria were determined via pretransplant diagnostic imaging, which is reported to UNOS by a given transplant center prior to OLT as part of ongoing patient registration.

### 2.2. Study Population

From the UNOS registry, a total of 3,434 patients underwent OLT for HCC during the study period. After excluding patients with missing tumor size or number, patients exceeding UCSF criteria, and patients <18 years of age, we obtained our final study cohort (*n* = 1, 972). These patients underwent OLT for HCC within Milan criteria (*n* = 1, 913) or outside of Milan criteria but within UCSF criteria (*n* = 59). Milan criteria were defined as 1 tumor ≤5 cm; or ≤3 tumors with each tumor ≤3 cm [8]. UCSF criteria were defined as 1 tumor ≤6.5 cm or ≤3 tumors with the largest tumor diameter ≤4.5 cm and total tumor diameter ≤8 cm [9]. Total tumor diameter was calculated as the sum of all hepatomas. Comorbidities of the study cohorts included diabetes mellitus (DM), body mass index (BMI), hepatitis B virus (HBV), and hepatitis C virus (HCV). The causes of death included malignancy (i.e., graft failure from recurrent disease or malignancy not otherwise specified) and other causes (i.e., cardiovascular, graft failure, multisystem organ failure, hemorrhage, infectious, and other). Liver-directed therapy (LDT) included transarterial chemoembolization (TACE), radiofrequency ablation (RFA), and cryoablation.

### 2.3. Statistical Analysis

The primary prognostic factor of interest was transplant criteria (i.e., Milan versus UCSF) and the association of this variable with survival. After patients were stratified by transplant criteria, the overall survival was calculated from the date of transplant to the date of death using the Kaplan-Meier method. The log-rank test was used to compare survival curves. Among the demographic and clinical data that were compared, age, BMI, and total tumor diameter were coded as continuous variables, whereas gender, DM, tumor number, LDT, HBV and HCV status, and cause of death were coded as categorical variables. Student's *t*-test and *χ* ^2^-test were used to calculate the differences in the continuous and categorical factors, respectively. Fisher's exact test was used for comparison of categorical variables, where appropriate. Univariate Cox regression analysis was performed to determine the association of each clinicopathologic factor with survival. Multivariate Cox regression analysis was applied to assess the association of multiple covariate factors with survival in the two transplant criteria cohorts, while controlling for the factors found to be significant on univariate analysis. Results were presented as hazard ratios (HR) and reported with 95% confidence intervals (CI) and two-sided *P* values. All statistical tests were considered significant when the corresponding *P* values were <0.05. SPSS (version 12.0, SPSS Inc., Chicago, IL, USA) was used to perform statistical analyses.

## 3. Results

### 3.1. Characteristics of the Patient Cohort

The entire study cohort consisted of 1,972 HCC patients who were transplanted within Milan or UCSF criteria from 2002 to 2007 (Table 1). The majority of patients (*n* = 1913; 97%) were within Milan criteria at the time of OLT based on imaging, while only 59 patients, or 3% of the total, underwent OLT with tumors that were beyond Milan criteria but within UCSF criteria. The majority of patients (79%) was male and had 1 tumor (67%). Only a small percentage of patients (24%) had DM, and most patients (69%) were nonobese. Local tumor control or downsizing with LDT was performed in 36% of patients. Causes of death included malignancy (31%) and all other causes (56%).

### 3.2. Comparison of Characteristics by Transplant Criteria

Patients who underwent OLT for HCC within Milan criteria and UCSF criteria were compared, as shown in Table 2. The majority of the Milan criteria cohort had 1 tumor (69%), whereas the majority of the UCSF criteria cohort had 1 tumor (39%) or 2 tumors (44%) (*P* < 0.001). LDT was also more frequently performed in the UCSF cohort (61% versus 35%, respectively, *P* < 0.001). Age, gender, DM, BMI, HBV status, HCV status, and cause of death were similar between the two cohorts.

### 3.3. Survival Analysis

**Figure 1:**
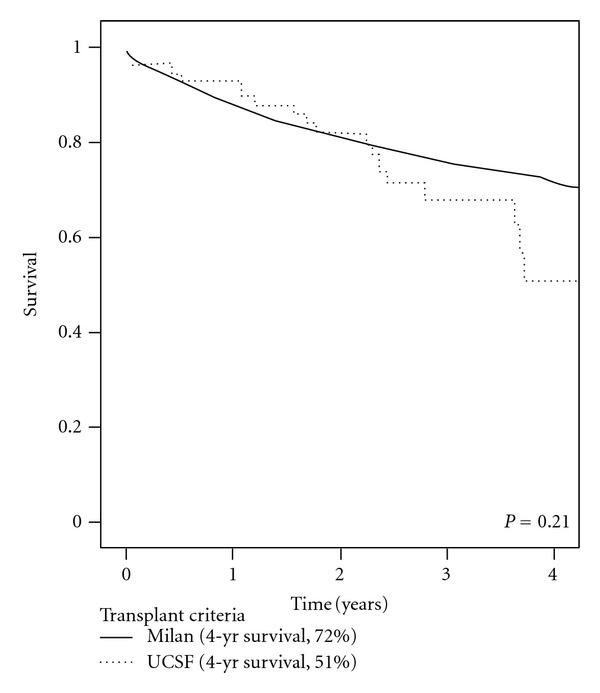
Survival analysis of HCC patients treated with liver transplantation stratified by Milan and UCSF selection criteria.

Kaplan-Meier curves were constructed to determine survival for the Milan and UCSF criteria cohorts (Figure 1). Survival between the Milan and UCSF criteria cohorts was similar (1-, 2-, 3- and 4-year survival rates: 89%, 81%, 76%, and 72% versus 91%, 80%, 68, and 51%, respectively, *P* = 0.21). By univariate and multivariate analysis, neither Milan nor UCSF criteria were independently predictive of improved survival (Table 3).

## 4. Discussion

 Liver transplantation remains a preferred treatment for patients with early-stage HCC in the setting of cirrhosis. Based on the accumulation of data showing excellent disease-free survival in patients with early-stage HCC treated by transplantation, the current United States allocation system for liver transplantation has given priority to patients with HCC within Milan criteria. With the incidence of HCC continuing to rise despite a relatively static organ supply, the allocation system requires continuing refinement so that patient benefit and outcome from this scarce resource are optimized. The current accepted Milan criteria for transplantation originally demonstrated 4-year survival and recurrence-free rates of 75% and 83%, respectively [8]. These results have been validated by numerous subsequent studies showing equivalent or superior survival advantages [10–13].

 In 2001, Yao et al. proposed expanding the current selection criteria. Referred to as the UCSF criteria, these expanded HCC transplantation criteria resulted in a modest increase in the total number of eligible patients of approximately 5–10% [9]. Using this single institution criteria, Yao et al. demonstrated a 5-year survival of 75% after OLT for HCC as compared to a 50% 1-year survival in patients who exceeded these criteria [9]. Their criteria were retrospectively determined by explant pathologic evaluation. These encouraging results were corroborated in a larger single institutional series that included 185 patients transplanted for HCC who also met UCSF criteria and exceeded Milan criteria. In that series, Duffy et al. reported a 5-year survival of 64% in patients beyond Milan but within UCSF criteria compared to a 79% 5-year survival for patients transplanted within Milan criteria (*P* = 0.061) [12]. Given the equivalent survival in patients transplanted within these expanded criteria, interest in revising the current organ allocation system has grown albeit with considerable caution and controversy.

Opponents of selection criteria expansion have suggested that the UCSF criteria are applicable to only a small subset of patients and cannot be applied in the pretransplant setting [5, 13]. Decaens et al. attempted to evaluate the UCSF criteria in a multiinstitutional setting. Pooling data from 14 French transplant centers, they identified 39 out of 461 patients who were transplanted beyond Milan criteria but within UCSF criteria, and compared these patients to 184 patients within Milan criteria. While survival was equivalent between the two groups when the criteria were applied to explant pathologic evaluation (64% versus 70%, respectively, *P* = 0.33), the 5-year survival in patients outside Milan but meeting UCSF criteria when applied to pretransplant diagnostic imaging was only 46%. Despite no statistical difference in survival between the two criteria groups, the authors suggested that the application of these criteria may be imprecise when used in pretreatment patient selection [13]. These concerns have been outlined by other series as well [15, 16]. Subsequently, Yao et al. reported another series of 38 patients exceeding Milan criteria but within UCSF criteria based on pretreatment diagnostic imaging and compared them to patients within pretreatment Milan criteria. There were no differences in survival, nor were there any differences in other risk factors for posttransplant recurrence, such as vascular invasion or poorly differentiated pathology between groups [17]. Similarly, Duffy et al. also did not identify a survival difference when Milan and UCSF criteria were compared according to pretransplant diagnostic imaging [12]. Overall these studies suggest that in a few select centers, pretransplant imaging is a reasonably accurate predictor of explant pathologic status, and that patients undergoing OLT for HCC have similar outcomes whether pretransplant imaging is within Milan or UCSF criteria.

Since the majority of evidence supporting the adoption of UCSF criteria comes from single institution series, we sought to evaluate these criteria within a multiinstitutional database. By using the UNOS database, we identified 59 patients transplanted for HCC within UCSF criteria, as compared to 1,913 patients transplanted within Milan criteria. We were unable to identify a survival difference between selection groups nor was UCSF criteria an independent predictor of worse survival on multivariate analysis. Importantly, patients within UCSF criteria were more likely to receive LDT when compared to patients within Milan criteria. Indeed, other single institution series have shown the benefit of downstaging via LDT in order to meet criteria, or to control disease while awaiting transplantation [18, 19]. Our results are not able to separately address the outcomes of patients who are initially outside of UCSF criteria on pretransplant imaging and subsequently undergo downstaging procedures to allow OLT. Additionally, due to the limitations of the database, information concerning the time interval from diagnosis to transplant and drop-out from the waiting list could not be ascertained.

The results of our study need to be carefully considered in light of our small patient cohort. Similar to other reported series, the number of patients in our study transplanted within UCSF criteria was small. However, the number of patients in our series beyond Milan criteria but within UCSF criteria (*n* = 59) compares favorably with Yao's two initial reports first establishing the UCSF criteria. Yao's initial series based on explant staging identified 18 patients meeting criteria and his subsequent analysis based on pretransplant imaging was based on 38 patients meeting UCSF criteria [9, 17]. However, given that the UNOS database covers nationwide transplant center reporting, we believe our findings may hold more weight in comparison to single institution series. Moreover, this is the largest multiinstitutional series comparing these two selection criteria within the United States patient population in the reported literature and the first time that the UNOS database has provided a comparison of transplantation outcomes for HCC based on selection criteria.

Before adopting an expanded size based criteria into the current UNOS allocation scheme, several questions require consideration. First, the ability to adequately stage patients with HCC remains a challenge. With current imaging modalities, understaging can be expected in 20–30% of patients, while overstaging can be seen in up to 15% of patients [20]. Given survival in patients with HCC beyond UCSF criteria is clearly inferior to survival within either Milan or UCSF criteria, further refinement of pretransplant staging is necessary to ensure size criteria validation. Furthermore, while size-based criteria may be comparable and reproducible in pretreatment staging, they may not predict the biologic aggressiveness of the underlying HCC. Recent evaluation into expanded criteria both from the University of Pittsburgh and a European multicenter series suggests that pathologic characteristics, in particular tumor grade and microvascular invasion, may be more important determinants of recurrence-free survival than size criteria [11, 21, 22]. Additionally, biopsy samples provide the opportunity for genetic profiling and molecular analysis, which may shed further insight into the tumor's biology [14]. However, obtaining adequate pretransplant tissue for pathologic evaluation in patients with active hepatitis or cirrhosis is not without inherent risk and thus limits the utility of such staging systems. Yao et al. tested the UCSF criteria to predict the Pittsburgh modified TNM staging system, and the results were favorable without the need for a pretransplant biopsy [21]. UNOS selection criteria are currently based solely on pretransplant imaging. Our preliminary results suggest that pretransplant imaging, while imperfect, may not negatively affect outcomes when used to stratify patients by UCSF or Milan criteria. Nonetheless, we acknowledge that our conclusions are limited by the constraints of the UNOS dataset. Lack of information on biopsy results and other pretransplant features, as well as the possibility of unknown confounding factors, may have influenced our results and affects the generalization of our findings [23, 24].

Perhaps the most difficult question to address is how the addition of candidates for transplantation will affect the overall survival of HCC patients listed for transplantation based on an intention-to-treat analysis. Current population based analyses and single institution series suggest drop-out rates of 12–18% secondary to tumor progression, while the intention to treat overall survival of all listed patients is approximately 50% [22, 25]. Patients exceeding Milan criteria do not receive prioritization in the current UNOS allocation system, thus limiting the ability to compare these two selection criteria objectively. Given that population prediction models anticipate a 20% increase in candidates for transplantation with adoption of the UCSF criteria, the adoption of expanded criteria must be weighed heavily against the limited organ supply [26]. However, when one considers that the drop-off rate increases to 30–40% when Milan criteria are used as absolute criteria, further refinement seems warranted [22, 25].

## 5. Conclusion

 In this largest, multiinstitutional series comparing UCSF to Milan criteria, we identified no difference in survival between patients transplanted by either selection criteria. Given the superior results of transplantation for appropriate patients with HCC and advanced liver disease, long-term prospective comparison of these two selection criteria appears warranted.

## Figures and Tables

**Table 1:** Characteristics of patients within the cohort.

Clinicopathologic factors	*N* = 1972
Age (yrs)	
Mean ± SD (Range)	56 ± 8 (18–78)
≤50	441 (22)
51–64	1211 (61%)
≥65	337 (17%)
Gender	
Female	418 (21%)
Male	1571 (79%)
Diabetes mellitus	
No	1475 (74%)
Yes*	481 (24%)
Unknown	33 (2%)
BMI	
Mean, ±SD	28.1 (4.9)
Range	(13–51)
<30	1380 (69%)
≥30	596 (30%)
Unknown	13 (1%)
Hepatitis B Virus	
Negative	1111 (56%)
Positive	662 (33%)
Unknown	216 (11%)
Hepatitis C Virus	
Negative	639 (33%)
Positive	1130 (57%)
Unknown	220 (11%)
Number of tumors	
Mean (range)	1 ± 0.7 (1–5)
1	1343 (67%)
2	452 (23%)
3	191 (10%)
4	2 (0%)
5	1 (0%)
Total Tumor Diameter (cm)	
Mean ± SD (range)	3 ± 1 (0.2–17)
Vital status	
Alive	1479 (74%)
Dead	510 (26%)
COD	
Malignancy recurrence	160 (31%)
Other	284 (56%)
Unknown	66 (13%)
Liver Directed Therapy^†^	
No	1274 (64%)
Yes	715 (36%)
Transplant criteria	
Milan	1913 (96%)
UCSF	59 (3%)

*Diabetes mellitus Type I or II; or type unknown.

^†^Radiofrequency ablation, transarterial chemoembolization, cryoablation.

BMI: body mass index; COD: cause of death.

**Table 2:** Comparison of patient characteristics by transplant criteria.

Factors	Transplant criteria		
	Milan	UCSF	*P* value
	(*N* = 1913 )	(*N* = 59)	
Age (yrs)			
Mean, ± SEM	56.2 ± 0.18	58.4 ± 1.02	
≤50	426 (22%)	11 (19%)	0.11
51–64	1169 (61%)	32 (54%)	
≥65	318 (17%)	16 (27%)	
Gender			0.87
Female	399 (21%)	13 (22%)	
Male	1514 (79%)	46 (78%)	
Diabetes mellitus			0.47
No	1418 (75%)	43 (77%)	
Yes*	465 (25%)	13 (23%)	
BMI			0.59
Mean, ±SEM	28.1 ± 0.11	28.4 ± 0.66	
Liver directed therapy			<0.001
No	1243 (65%)	23 (39%)	
Yes	670 (35%)	36 (61%)	
Number of tumors			<0.001
1	1316 (69%)	23 (39%)	
2	420 (22%)	26 (44%)	
3	177 (9%)	10 (17%)	
Vital status			0.37
Alive	1426 (74%)	41 (70%)	
Dead	487 (26%)	18 (30%)	
Hepatitis C Virus			0.45
Negative	615 (36%)	20 (42%)	
Positive	1094 (64%)	28 (58%)	
Hepatitis B Virus			0.88
Negative	1073 (63%)	30 (61%)	
Positive	639 (37%)	19 (39%)	
Total Tumor Diameter			<0.001
Mean ± SEM	3.27 ± 0.03	5.86 ± 0.09	
COD			0.01
Malignancy recurrence	149 (35%)	9 (69%)	
Other	278 (65%)	4 (31%)	

*Yes: type I or II; or type unknown.

SEM: standard error of the mean; BMI: body mass index; COD: cause of death.

**Table 3:** Univariate and multivariate analysis.

Factors	HR (95% CI)	*P*	HR (95% CI)	*P*
Age	1.02 (1.01–1.03)	0.005	1.02 (1.01–1.03)	0.002
Gender		0.03		0.11
Female	1.0	—	1.0	—
Male	0.80 (0.65–0.98)	0.03	1.20 (0.96–1.49)	0.11
Diabetes mellitus		0.29		
No*	1.0	—	N/A	N/A
Yes	1.11 (0.91–1.36)	0.29		
BMI	1.00 (0.98–1.02)	0.72	N/A	N/A
Number of tumors		0.78		
1	1.0	—		
2	0.95 (0.77–1.17)	0.63	N/A	N/A
3	0.91 (0.67–1.24)	0.55		
Total Tumor Diameter	1.08 (1.01–1.16)	0.02	1.09 (1.01–1.18)	0.03
Liver directed therapy		0.04		0.06
No	1.0	—	1.0	—
Yes	0.82 (0.67–0.99)	0.04	1.22 (0.99–1.50)	0.06
Hepatitis B virus		0.47		
Negative	1.0	—	N/A	N/A
Positive	0.93 (0.77–1.13)	0.47		
Hepatitis C virus		0.02		0.004
Negative	1.0	—	1.0	—
Positive	1.26 (1.03–1.54)	0.02	1.34 (1.10–1.64)	0.004
Transplant criteria		0.22		0.32
Milan	1.0	—	1.0	—
UCSF	1.34 (0.84–2.15)	0.22	1.31 (0.77–2.21)	0.32

*Yes: type I or II; or type unknown.

HR: hazard ratio; CI: confidence interval; BMI: body mass index.
